# Comparison of two portable clinical analyzers to one stationary analyzer for the determination of blood gas partial pressures and blood electrolyte concentrations in horses

**DOI:** 10.1371/journal.pone.0211104

**Published:** 2019-02-15

**Authors:** Katharina Kirsch, Johann Detilleux, Didier Serteyn, Charlotte Sandersen

**Affiliations:** 1 Equine Clinic, Department of Companion Animals and Equids, Faculty of Veterinary Medicine, University of Liège, Liège, Belgium; 2 Department of Quantitative Genetics, Faculty of Veterinary Medicine, University of Liège, Liège, Belgium; University College Dublin, School of Veterinary Medicine, IRELAND

## Abstract

Portable blood gas analyzers are used to facilitate diagnosis and treatment of disorders related to disturbances of acid-base and electrolyte balance in the ambulatory care of equine patients. The aim of this study was to determine whether 2 portable analyzers produce results in agreement with a stationary analyzer. Blood samples from 23 horses hospitalized for various medical reasons were included in this prospective study. Blood gas analysis and electrolyte concentrations measured by the portable analyzers VetStat and epoc were compared to those produced by the cobas b 123 analyzer via concordance analysis, Passing-Bablok regression and Bland-Altman analysis. Limits of agreement indicated relevant bias between the VetStat and cobas b 123 for partial pressure of oxygen (pO_2_; 27.5–33.8 mmHg), sodium ([Na^+^]; 4.3–21.6 mmol/L) and chloride concentration ([Cl^-^]; 0.3–7.9 mmol/L) and between the epoc and cobas b 123 for pH (0.070–0.022), partial pressure of carbon dioxide (pCO_2_; 3.6–7.3 mmHg), pO_2_ (36.2–32.7 mmHg) and [Na^+^] (0.38.1 mmol/L). The VetStat analyzer yielded results that were in agreement with the cobas b 123 analyzer for determination of pH, pCO_2_, bicarbonate ([HCO_3_^-^]) and potassium concentration [K^+^], while the epoc analyzer achieved acceptable agreement for [HCO_3_^-^] and [K^+^]. The VetStat analyzer may be useful in performing blood gas analysis in equine samples but analysis of [Na^+^], [Cl^-^] and pO_2_ should be interpreted with caution. The epoc delivered reliable results for [HCO_3_^-^] and [K^+^], while results for pH, pCO2, pO2 and [Na^+^] should be interpreted with caution.

## Introduction

Awareness of acid-base and/or electrolyte abnormalities in equine medicine influence the choice of treatment and prognosis [1], with frequent monitoring of acid-base and electrolyte values in horses allowing early detection and intervention. Arterial blood gas analysis enables the assessment of pulmonary oxygenation efficiency and ventilation status [2], which may be of clinical value for the monitoring of patients under general anesthesia, patients with thoracic or respiratory diseases as well as critically ill neonates [3]. Assessment of acid-base and electrolyte concentration also provides useful information for the diagnosis and management of metabolic disorders in critically ill horses [3], horses with gastrointestinal disorders [1] and hereditary conditions (e.g., hyperkalemic periodic paralysis, recurrent exertional rhabdomyolysis) in which alterations of blood electrolyte concentrations are commonly observed [4]. Monitoring of acid-base and electrolyte status in exercising horses is also of clinical use with horses undertaking high-intensity exercise such as racing, eventing or endurance competitions often developing alterations in acid-base and electrolyte status [5–12] leading to disorders such as exercise-induced dehydration, cardiac dysrhythmias, laminitis or exertional rhabdomyolysis [13]. The utilization of portable Point-of-Care (POC) devices provides immediate measurement results for stall-side testing in the clinic and in a field setting. However, not all POC devices give comparable results as shown by a recent study comparing 2 POC blood gas analyzers to a stationary one in horses [14]. In this study it was revealed that performance of the POC devices was good for some parameters, while insufficient for other parameters [14]. Thus, the purpose of the present study was to determine whether 2 POC blood gas analyzers, the VetStat (Idexx Laboratories, Westbrook, USA) and the epoc (Alere, Waltham, USA), produce results in agreement with a stationary blood gas analyzer, the cobas b 123 (Pont-of-Care system, Roche, Basel, Switzerland) for the measurement of pH, partial pressure of carbon dioxide (pCO_2_) partial pressure of oxygen (pO_2_), bicarbonate (HCO_3_^-^), sodium (Na^+^), potassium (K^+^) and chloride (Cl^-^) concentrations in arterial and venous equine blood samples.

## Material and methods

### Animals

The study was approved by the institutional committee for the ethical use of animals (Commission Ethique Animale de Université de Liège). Owners of the horses gave informed consent for data collected from their horses to be used for scientific purposes. Blood samples from 23 horses admitted to the equine clinic of the University of Liège between May and June 2015 were included in this study. Patients with diverse medical conditions were included to represent a wide range of measured variables. Horses were sampled for diagnostic purposes only as determined to be necessary by a veterinary clinician unrelated to the present study.

### Instruments

Both the VetStat and epoc analyzers utilize disposable, single-use cartridges covering a range of analytical capabilities. The epoc analyzer requires a sample volume of 95 μL and takes less than 1 minute, while the VetStat analyzer requires a sample volume of 125 μl and takes less than 2 minutes for sample analysis.

The stationary POC system cobas b 123 and the POC analyzer epoc determine pH, pCO_2_ and electrolyte concentrations via a potentiometric method using ion selective electrodes and the Nernst-equation. The pO_2_ is measured via an amperometric method with ion-selective electrodes of the Clarke type. The POC blood gas analyzer VetStat determines the blood gas partial pressures and electrolyte concentrations by measuring their optical fluorescence using optodes. The [HCO_3_^-^] is calculated by means of the Henderson-Hasselbalch equation in all 3 instruments.

All analyzers were calibrated, maintained and operated according to their manufacturer’s instructions. All machines were kept at constant room temperature for at least 24 hours before and during the experiments. The epoc reader runs a calibration cycle for each cassette, containing an integrated quality control system with specific reference liquids for this machine. Each batch of VetStat sample cassettes is calibrated during the manufacturing process with the calibration information contained in the bar code that is scanned before each analysis. Internal calibration and quality control processes are performed by the reader with the VetStat reader calibrated with standard reference cassettes, standard hemoglobin cassettes and standard gas cartridges at prescribed intervals. The cobas b 123 system runs automated calibration cycles at defined intervals. The system calibration runs once a day in the morning with a 1 point-calibration running every 60 minutes, and a 2-point-calibration, running every 8 hours; these are postponed if the system starts a cycle during analyses.

### Sample collection

Arterial or venous blood samples were taken with pre-filled heparinized single-use disposable syringes (Monovette Lithium Heparin, Sarstedt, Germany) during standard examination of the horses or as part of the anesthetic monitoring. Venous blood samples were collected anaerobically by venipuncture of the jugular vein. Arterial blood samples were collected from the carotid or transverse facial artery. The patient’s rectal temperature was noted to allow for temperature correction.

### Sample analysis

Analysis was immediately performed after blood sample collection. Each blood sample was analyzed once by the stationary analyzer and the 2 POC analyzers, with the order of the analyzers pre-determined by an online randomization program (www.random.org). All measurements were undertaken by 1 of 2 people trained to use the equipment. The devices were situated adjacent to each other in order to minimize the time interval between measurements. A maximum of 5 minutes delay for introducing samples to all 3 analyzers was considered acceptable. In order to determine repeatability of the measurements with the three compared instruments, seven serial measurements of the same blood sample were performed on each analyzer. The procedure of these serial measurements was conducted as fast as possible within a time range less than 25 minutes in order to minimize the changes in sample composition over time.

### Statistical analysis

Concordance correlation analysis, Passing-Bablok regression analysis and the Bland-Altman method (MedCalc Statistical Software version 16.1, Ostend, Belgium) were performed to determine agreement between the 3 analyzers. Lin’s concordance correlation coefficient (r_ccc_) contains a measure of precision (Pearson’s ρ) and accuracy (bias corrected factor, C_b_) and was used as an indicator for the strength of concordance between measurements [15]. A value of ≤ 0.90 was considered to be poor agreement, 0.90–0.95 as moderate agreement, 0.95–0.99 as substantial agreement and ≥0.99 as almost perfect agreement between methods [16].

The intercept of the Passing-Bablok regression equation reflects the constant bias, the slope reflects the proportional bias and the residual standard deviation (RSD) reflects the random bias. Confidence intervals reveal if these values differ from value 0 for intercept and from value 1 for slope only by chance. Therefore, if the 95% CI includes the value 0 for the intercept it can be assumed that there is no significant difference between the obtained intercept value and the value 0 and therefore no constant bias between the 2 methods. For the slope, a 95% CI including the value 1 means that there is no significant difference between the obtained slope value and the value 1 and that there is no significant proportional bias between two methods [17]. Assuming normal distribution, 95% of random differences are expected to lie within ± 1.96 RSD, with a large interval considered to indicate a high imprecision [17]. To warrant the validity of the Passing-Bablok procedure, the linear relationship between compared results was assessed by the cusum test.

In addition to regression analysis, Bland-Altman plots were prepared by plotting the difference between 2 compared measurements against the mean of the 2 measurements. The mean difference between results was considered to reflect the systematic bias. If the 95% confidence interval of the bias contained the value 0, no significant systematic bias between methods was assumed [18,19]. Provided that differences between measurements are normally distributed, 95% of the data points should lie within 1.96 SD of the mean difference. This assumption allowed the calculation of 95% limits of agreement [17,18]. The limits of agreement served as estimation of the total bias consisting of systematic and random bias and were assessed by comparing them to pre-set clinical allowable errors. The bias was assumed as clinically relevant if the 95% limits of agreement ranged outside the limits for the allowable total errors. The assignment of limits for clinical acceptance were based on the CLIA guidelines [20,21].

Repeatability for each analyzer was assessed using the mean, the within-run standard deviation (SD_w-run_) and the within-run coefficient of variation in percent (CV_w-run_). The clinical evaluation of the obtained precision results was carried out by comparing them to pre-set clinical limits of acceptance with the specification that they should be a quarter or less of the defined allowable total error [21, 22].

The underlying assumptions of normal distribution was checked by visual inspection of the normal probability plots for the differences between compared results. Statistical significance was set at P ≤ 0.05.

## Results

The study group was composed of 4 stallions, 6 geldings and 13 females, aged between 2 months and 25 years.

A total of 41 blood samples (1 to 4 samples per horse) including 32 arterial and 9 venous samples were collected and analyzed. Due to cartridge calibration failures, 8 epoc results and 2 VetStat results were incomplete. Furthermore, 3 results for [K^+^] from the epoc analyzer failed to be recorded. Therefore, 39 complete data sets were compared between the VetStat and cobas b 123 and 33 complete data sets between the epoc and cobas b 123 (30 data sets for [K^+^]). Results for [Cl^-^] were only compared between VetStat and cobas b 123 since the epoc cartridges do not provide [Cl^-^] values. The differences between compared results were normally distributed.

Table 1 summarizes results of concordance correlation analysis for the comparison between the cobas b 123 and VetStat and the cobas b 123 and epoc analyzers.

**Table 1:** Concordance correlation analysis between the portable analyzers VetStat (*n* = 39; 30 arterial, 9 venous) and epoc (*n* = 33; 24 arterial, 9 venous) and the cobas b 123 analyzer. Lin’s concordance correlation coefficients (r_ccc_) with 95% confidence intervals (95% CI), precision (Pearson ρ) and accuracy (bias correction factor, C_b_) are reported.

Measured variables		n	Concordance correlation analysis		
			Lin’s r_ccc_ (95% CI)	Precision ρ	Accuracy C_b_
pH	cobas/VetStat	39	0.954 (0.920 to 0.974)	0.973	0.981
	cobas/epoc	33	0.870 (0.782 to 0.924)	0.962	0.920
pCO_2_ (mmHg)	cobas/VetStat	39	0.968 (0.939 to 0.983)	0.954	0.912
	cobas/epoc	33	0.934 (0.878 to 0.965)	0.959	0.974
pO_2_ (mmHg)	cobas/VetStat	39	0.991 (0.984 to 0.995)	0.992	0.999
	cobas/epoc	33	0.985 (0.972 to 0.993)	0.987	0.998
HCO_3_^-^(mmol/L)	cobas/VetStat	39	0.846 (0.736 to 0.913)	0.892	0.949
	cobas/epoc	33	0.862 (0.743 to 0.928)	0.867	0.994
Na^+^ (mmol/L)	cobas/VetStat	39	0.083 (0.022 to 0.143)	0.471	0.175
	cobas/epoc	33	0.738 (0.602 to 0.833)	0.941	0.785
K^+^ (mmol/L)	cobas/VetStat	39	0.965 (0.935 to 0.981)	0.974	0.990
	cobas/epoc	30	0.951 (0.905 to 0.976)	0.970	0.981
Cl^-^ (mmol/L)	cobas/VetStat	39	0.363 (0.220 to 0.491)	0.772	0.470

**Fig 1:**
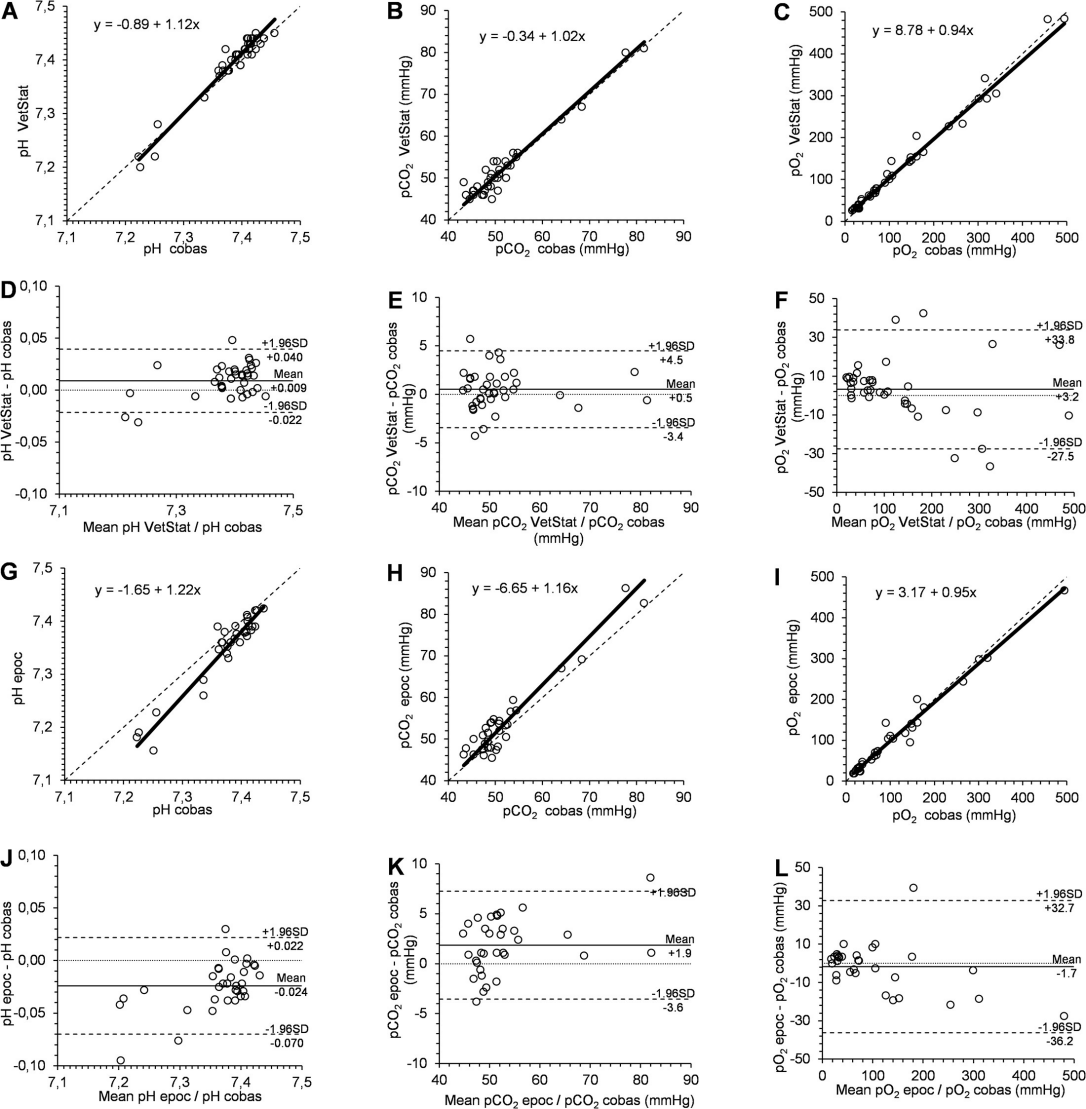
Passing-Bablok regression analysis and Bland-Altman plot analysis for comparison of different analyzers for blood pH, pCO_2_, and pO_2_.

**Fig 2:**
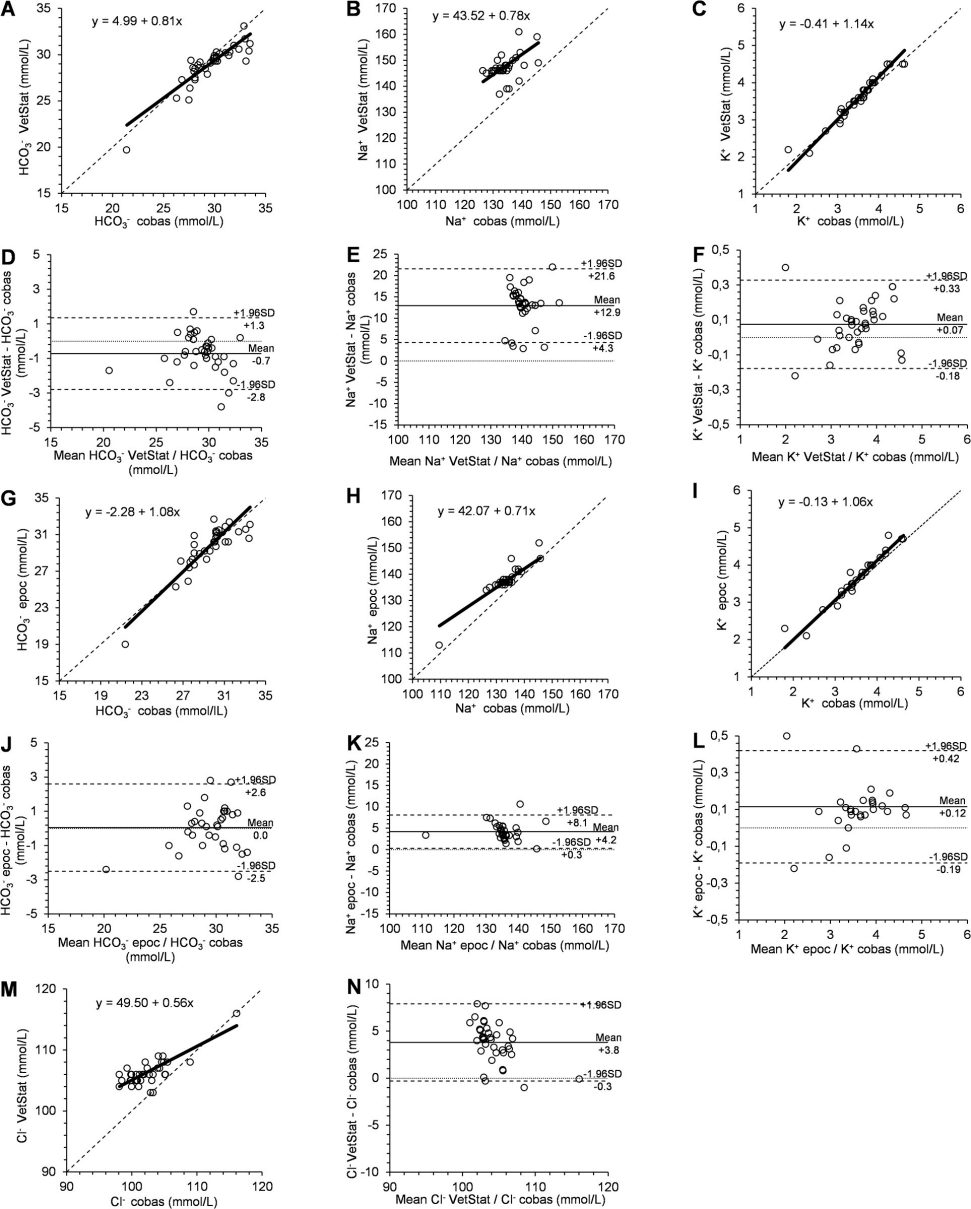
Passing-Bablok regression analysis and Bland-Altman plot analysis for comparison of different analyzers for blood [HCO3-], [Na^+^], [K^+^] and [Cl^-^]. Passing-Bablok regression analysis for comparison of VetStat versus the cobas b 123 analyzer for blood [HCO_3_^-^] (A), [Na^+^] (B), [K^+^] (C), and [Na^+^] (M), and for comparison of epoc versus cobas b 123 analyzer for blood [HCO_3_^-^] (G), [Na^+^] (H) and [K^+^] (I). The dashed line is the line of identity, the solid line is the best fit. Bland-Altman plot for comparison of VetStat versus cobas b 123 analyzer for blood [HCO_3_^-^] (D), [Na^+^] (E), [K^+^] (F) and [Cl^-^] (N) and for comparison of epoc versus cobas b 123 analyzer for blood [HCO_3_^-^] (J), [Na^+^] (K), and [K^+^] (L). The dashed lines are upper and lower limit of agreement, the solid line is the mean difference (bias).

In Table 2, the results of the Passing-Bablok regression analysis and the Bland-Altman plots from the comparison between analyzers are displayed for each parameter. Figs 1 and 2 show the Passing-Bablok regression analysis and the Bland-Altman plots for the comparison between the VetStat and the cobas b 123 analyzer and between the epoc and the cobas b 123 analyzer for pH, pCO_2_, pO_2,_ and for [HCO_3_^-^], [Na^+^], [K^+^] and [Cl^-^], respectively.. The cusum test indicated no significant deviation from linear relationship between compared results for all determined parameters (P > 0.05).

**Table 2:** Intercept, slope and residual standard deviation of Passing-Bablok regression as well as bias, lower and upper limit of agreement from the Bland-Altman plots and their 95% confidence intervals (95% CI) from the comparisons between the cobas b 123 and VetStat and the cobas b 123 and epoc analyzers.

Measured variables		Passing-Bablok regression			Bland-Altman plot		
		Intercept (95% CI)	Slope (95% CI)	Residual standard deviation (95% CI)	Bias (95% CI)	Lower limit of agreement (95% CI)	Upper limit of agreement (95% CI)
pH	cobas/ VetStat	-0.89 (-1.84 to 0.13)	1.12 (0.98 to 1.25)	0.010 (-0.019 to 0.019)	0.009 (0.004 to 0.014)	-0.022 (-0.030 to -0.013)	0.040 (0.031 to 0.048)
	cobas/ epoc	-1.65 (-3.55 to -0.41)	1.22 (1.05 to 1.48)	0.014 (-0.027 to 0.027)	-0.024 (-0.032 to -0.016)	-0.070 (-0.084 to -0.056)	0.022 (0.007 to 0.036)
pCO_2_ (mmHg)	cobas/ VetStat	-0.34 (-7.51 to 5.18)	1.02 (0.91 to 1.15)	1.450 (-2.841 to 2.841)	0.5 (-0.1 to 1.2)	-3.4 (-4.6 to -2.3)	4.5 (3.4 to 5.6)
	cobas/ epoc	-6.65 (-20.31 to 1.10)	1.16 (1.00 to 1.44)	1.885 (-3.695 to 3.695)	1.9 (0.9 to 2.8)	-3.6 (-5.3 to -1.9)	7.3 (5.6 to 9.0)
pO_2_ (mmHg)	cobas/ VetStat	8.78 (4.80 to 11.09)	0.94 (0.90 to 0.97)	11.425 (-22.393 to 22.393)	3.2 (-1.9 to 8.3)	-27.5 (-36.2 to -18.7)	33.8 (25.1 to 42.6)
	cobas/epoc	3.17 (1.01 to 6.21)	0.95 (0.91 to 1.01)	12.019 (-23.557 to 23.557)	-1.7 (-8.0 to 4.5)	-36.2 (-47.0 to -25.5)	32.7 (22.0 to 43.5)
HCO_3_^-^ (mmol/L)	cobas/ VetStat	4.99 (-0.60 to 10.80)	0.81 (0.62 to 1.00)	0.787 (-1.543 to 1.543)	-0.7 (-1.1 to -0.4)	-2.8 (-3.4 to -2.2)	1.3 (0.8 to 1.9)
	cobas/ epoc	-2.28 (-10.38 to 6.21)	1.08 (0.79 to 1.36)	0.918 (-1.800 to 1.800)	0.0 (-0.4 to 0.5)	-2.5 (-3.3 to -1.7)	2.6 (1.8 to 3.4)
Na^+^ (mmol/L)	cobas/ VetStat	43.52 (6.26 to 85.21)	0.78 (0.47 to 1.05)	3.277 (-6.423 to 6.423)	12.9 (11.5 to 14.4)	4.3 (1.8 to 6.8)	21.6 (19.1 to 24.0)
	cobas/ epoc	42.07 (15.73 to 66.07)	0.71 (0.54 to 0.91)	1.921 (-3.765 to 3.765)	4.2 (3.5 to 4.9)	0.3 (-0.9 to 1.5)	8.1 (6.9 to 9.3)
K^+^ (mmol/L)	cobas/ VetStat	-0.41 (-0.71 to 0.00)		0.097 (-0.191 to 0.191)	0.07 (0.03 to 0.12)	-0.18 (-0.25 to -0.11)	0.33 (0.25 to 0.40)
	cobas/ epoc	-0.13 (-0.49 to 0.10)	1.14 (1.02 to 1.22)	0.110 (-0.216 to 0.216)	0.12 (0.06 to 0.17)	-0.19 (-0.29 to -0.09)	0.42 (0.32 to 0.52)
Cl^-^ (mmol/L)	cobas/ VetStat	49.50 (30.02 to 68.26)	0.56 (0.36 to 0.74)	1.242 (-2.434 to 2.434)	3.8 (3.1 to 4.5)	-0.3 (-1.5 to 0.9)	7.9 (6.7 to 9.1)

Passing-Bablok regression analysis for comparison of VetStat versus cobas b 123 analyzer for blood pH (A), pCO_2_ (B), and pO_2_ (C) and for comparison of epoc versus cobas b 123 analyzer for blood pH (G), pCO_2_ (H), and pO_2_ (I). The dashed line is the line of identity, the solid line is the best fit.

Bland-Altman plot analysis for comparison of VetStat versus cobas b 123 analyzer for blood pH (D), pCO_2_ (E), and pO_2_ (F) and for comparison of epoc versus cobas b 123 analyzer for blood pH (J), pCO_2_ (K), and pO_2_ (L). The dashed lines are upper and lower limit of agreement, the solid line is the mean difference (Bias).

### pH

For the determination of pH, Bland-Altman analysis indicated a significant positive systematic bias between the VetStat and cobas b 123 analyzers (P = 0.0009). However, the estimated 95% limits of agreement indicated that the amount of systematic and random bias remained within the pre-set limits of acceptance. For pH determined by the epoc and the cobas b 123 analyzer, Bland-Altman analysis detected a significant negative systematic bias (P < 0.0001) while the 95% limits of agreement indicated that the underestimation of pH by the epoc analyzer ranged outside the limits of acceptance. The Passing-Bablok regression indicated a significant constant as well as proportional bias.

### pCO_2_

For the determination of pCO_2_, no significant systematic bias between the VetStat and cobas b 123 analyzers was detected with the 95% limits of agreement within the limits of acceptance. However, a significant positive systematic bias between the epoc and the cobas b 123 analyzer for the determination of pCO_2_ was detected (P = 0.0005). The 95% limits of agreement indicated that the epoc analyzer overestimated the pCO_2_ beyond the pre-set limits of acceptance.

### pO_2_

For the determination of pO_2_, no significant systematic bias between the VetStat and cobas b 123 analyzer as well as between the epoc and the cobas b 123 analyzer was identified. However, the 95% limits of agreement for both instruments indicated random deviations between the analyzers, which were higher than the pre-set limits of acceptance, especially for very high pO_2_ values. Due to the subjection of the magnitude of random error on the level of the pO_2_, the 95% limits of agreement were additionally calculated for pO_2_ values up to 100 mmHg for the comparison between the VetStat and the cobas b 123 analyzers (mean bias: 5.9 mmHg, 95% limits of agreement: -2.9 to +14.6 mmHg, *n* = 20) and between the epoc and the cobas b 123 analyzers (mean bias: 1.2 mmHg, 95% limits of agreement: -8.4 to +10.8 mmHg, *n* = 19). For pO_2_ values up to 100 mmHg, the 95% limits of agreement were considerably closer, although the random error for the determination of pO_2_, including exclusively lower values, still ranged outside the pre-set limits of acceptance.

### [HCO_3_^-^]

For the determination of [HCO_3_^-^], a significant negative systematic bias between the VetStat and cobas b 123 analyzers (P = 0.0001) was found. However, the 95% limits of agreement ranged within the limits of acceptance. For [HCO_3_^-^] calculated by the epoc and cobas b 123 analyzers, no significant systematic bias was found.

### [Na^+^]

For the determination of [Na^+^], significant positive systematic bias between the VetStat and cobas b 123 analyzers as well as between the epoc and cobas b 123 analyzers (P < 0.0001) was identified. The 95% limits of agreement ranged outside the pre-set limits of acceptance. Between the epoc and the cobas b 123 analyzers, significant constant as well as proportional bias was detected.

### [K^+^]

For the determination of [K^+^], significant positive systematic bias between the VetStat and the cobas analyzers (P = 0.0009) as well as between the epoc and the cobas b 123 analyzers (P = 0.0004) was found. The 95% limits of agreement remained within the pre-set limits of acceptance. Between the VetStat and the cobas b 123 analyzer, significant proportional bias was detected.

### [Cl^-^]

For the determination of [Cl^-^], significant positive systematic bias between the VetStat and the cobas b 123 analyzer (P < 0.0001) was found. The 95% limits of agreement indicated an overestimation of [Cl^-^] by the VetStat analyzer which ranged outside the pre-set limits of acceptance. Besides the constant bias, significant proportional bias was detected.

### Repeatability

Repeatability results for each analyzer are summarized in Table 3 with results compared to published [22]. The cobas b 123 analyzer failed to meet precision targets for pCO_2_ and [K^+^], the VetStat failed to meet precision targets for [Cl^-^], while the epoc failed to meet the precision targets for pCO_2_, pO_2_, and [K^+^].

**Table 3:** Mean, within-run standard deviation (SD_w-run_) and within-run coefficient of variation (CV_w-run_) in percent from one blood sample analyzed for each of the 3 analyzers (cobas b 123, VetStat and epoc) and compared to published precision targets [21].

Measured variables	cobas b 123		VetStat		epoc		
	Mean ± SD_w-run_	CV_w-run_ (%)	Mean ± SD_w-run_	CV_w-run_ (%)	Mean ± SD_w-run_	CV_w-run_ (%)	Precision target (CV%)
pH	7.386 ± 0.005	0.07	7.436 ± 0.011	0.15	7.341 ± 0.004	0.06	0.1[14,22]
pCO_2_ (mmHg)	42.71 ± 1.13	**2.64**	50.29 ± 0.49	0.97	52.11 ± 1.68	**3.22**	2.4[14,22]
pO_2_ (mmHg)	95.19 ± 3.88	4.08			314.39 ± 18.71	**5.95**	4.8[14,22]
HCO_3_^-^ (mmol/L)	25.00 ± 0.37	1.50	31.19 ± 0.48	1.53	28.19 ± 0.76	2.69	<5[14,22]
Na^+^ (mmol/L)	133.1 ± 1.0	0.70	149.4 ± 1.1	0.80	135.6 ± 1.4	1.00	0.3[14,22]
K^+^ (mmol/L)	4.040 ± 0.125	**3.09**	4.286 ± 0.038	0.88	4.114 ± 0.267	**6.50**	2.3[14,22]
Cl^-^ (mmol/L)	100.8 ± 0.2	0.20	111.0 ± 1.4	**1.30**			0.6[22]

pCO_2_, partial pressure of carbon dioxide; pO_2_, partial pressure of oxygen; HCO_3_^-^, bicarbonate; Na^+^, sodium; K^+^, potassium; Cl^-^, chloride. Values not meeting the precision targets are in bold.

## Discussion

Determination of blood gas partial pressures and electrolyte concentrations at stall-side and under field conditions requires that portable analyzers yield results in agreement to analyzers used in veterinary clinics or similar institutions. To verify whether this condition is met for the POC analyzers VetStat and epoc, their performances were compared to the stationary cobas b 123 analyzer. Even though the cobas b 123 analyzer does not have to be permanently installed and therefore may be considered a mobile unit, it is not specifically intended for field use.

By evaluating the types and magnitudes of the differences as compared with pre-set clinical limits of acceptance, decisions can be made about the acceptability of methods [23]. The decision whether the differences between methods are acceptable depends on the aim of the tests and is a question of clinical judgment [18,19]. Thus, the pre-set clinical limits of acceptance are not fixed and can differ from one application to another. In this study the assignment of limits for clinical acceptance were based on the CLIA guidelines [20,21].

Use of Pearson’s correlation coefficient as a sole measure of agreement between methods is not appropriate. Furthermore, high correlation does not necessarily mean agreement since the correlation coefficient cannot detect systematic bias [15,24,25]. Therefore, concordance correlation analysis is the preferred method to determine agreement between analyzers. Lin’s concordance correlation coefficient indicates the strength of the relationship between two measurements by determining the variation from the line of equality and therefore also accounts for systematic deviations between methods [15]. The Bland-Altman analysis enables the determination of systematic bias between compared methods by calculating the mean difference between measurement results [18,19]. The calculation of limits of agreement further allow the estimation of the total bias consisting of systematic and random bias. The comparison of these limits of agreement to pre-set clinical limits of acceptance enable an assessment of the clinical relevance of the determined bias.

Since measurement errors had to be assumed in both comparison and test methods, the Passing-Bablok regression was preferred over ordinary linear regression [17]. In contrast to ordinary linear regression, the result of Passing-Bablok regression does not depend on the assignment of the instruments as reference and test method [17]. However, the lack of a validated reference method remains the major limitation of the present study. We compared 2 POC units to a stationary one, and even though 1 of the POC units (the epoc analyzer) has been validated for the use in dogs and more recently in horses, the stationary analyzer has not undergone scrutinized validity and precision testing against a validated reference method. ICSH guidelines acknowledge that in general a new machine should be compared to an existing one, even if the existing machine has not undergone validity testing [26]. This however is far from ideal with values thus interpreted with caution.

Concordance correlation indicated an almost perfect agreement between the VetStat and the cobas b 123 analyzers for pO_2_, a substantial agreement for pH, pCO_2_ and [K^+^] and a poor agreement for [HCO_3_^-^], [Na^+^] and [Cl^-^]. The agreement between the epoc and the cobas b 123 analyzer was substantial for pO_2_ and [K^+^], moderate for pCO_2_ and poor for pH, [HCO_3_^-^] and [Na^+^]. Based on the reported 95% limits of agreement and their comparison to pre-set limits of clinical acceptance, the systematic bias between the VetStat and the cobas b 123 analyzer for the determination of [Na^+^] and [Cl^-^] was clinically relevant, with the [Na^+^] considerably overestimated by the VetStat analyzer. The identified proportional bias meant that the slight overestimation of [K^+^] by the VetStat analyzer would increase with increasing [K^+^], while the overestimation of [Cl^-^] would increase with decreasing [Cl^-^]. Systematic bias between the epoc and the cobas b 123 analyzers was found to be clinically relevant for pH, pCO_2_ and [Na^+^] and [K^+^], with the epoc analyzer underestimating the pH and overestimating pCO_2_ and [Na^+^]. The identified proportional bias means that the underestimation of the pH by the epoc analyzer would increase with decreasing pH values, while the overestimation of [Na^+^] would increase with decreasing [Na^+^]. Since the determination of [Cl^-^] is not provided by the epoc analyzer, this parameter could not be included in the comparison.

Although no significant systematic bias was detected for the determination of pO_2_, the 95% limits of agreement for both analyzers indicated a high random error ranging outside clinically acceptable limits, especially for high pO_2_ values.

The results of the present study are comparable to results of previous studies evaluating epoc and VetStat analyzers with other POC or stationary analyzers. One study evaluating the epoc and a second POC analyzer (i-STAT) in dogs revealed that the precision of the epoc was acceptable, but agreement between the two methods was poor for measurement of [Na^+^], pH, pO_2_, hematocrit, hemoglobin and base excess (BE) [27]. A second study comparing the epoc and i-STAT to a stationary analyzer the ABL77 showed that agreement targets were not met for hemoglobin, hematocrit, and BE for the ABL77 and the i-STAT, while precision targets were not met with the epoc for pCO_2_, ionized calcium (iCa2^+^), hematocrit and hemoglobin [14]. Very recently, comparison of the epoc analyzer to a POC machine called Nova Biomedical Critical Care Xpress for equine and canine samples revealed varying degrees of bias, with good agreement between the two analyzers for p_a_O_2_, [Na^+^] and [K^+^] but less agreement for p_a_CO_2_ and [Cl^-^] [28]. In dogs, comparison of four machines (cobas b 123, IRMA TruPoint, VetStat, and ABL80 FLEX) revealed that pO_2_, pCO_2_, and pH to differ significantly between all four analyzers, with differences in pO_2_ substantial and clinically relevant [29]. The varying results for each of these different studies is likely due to differences in the analyzers assessed, clinical settings and statistical methodologies supporting the fact that validation of POC machines should not be used interchangeably.

There are numerous limitations in this study. Due to limited time and resources, the sample size for the present study was relatively small (*n* = 39), with each sample analyzed only once on each analyzer instead of in duplicate as recommended by CLSI guidelines. However, when adopting the null hypothesis that there is no difference between the analyzers, setting the alpha error at 0.05 and the (1-β) error at 0.8, the minimal sample size for a Cohen d effect size of 0.8 (P*power, University Düsseldorf) was determined to be n = 21. A small sample size was also a limitation in the repeatability analysis. Repeatability or within-run precision was measured by analysis of a single sample 7 consecutive times for each machine. Although the method chosen for the present study was similar to what is suggest by the ICSH [26], only 7 measurements were made in the same run instead of 10, due to limitations to the quantity of blood available based on the size of the syringe used to collect the blood. However, a recent study has demonstrated the within-laboratory precision of the epoc analyzer with equine blood based on measurement of 21 samples in duplicate [14]. As compared to published precision targets [22], the epoc analyzer met precision targets for pH, pO_2_, [Na^+^], [K^+^], glucose, lactate, [HCO_3_^-^], BE, and saturation of oxygen (SO_2_) while it failed to meet precision targets for pCO_2_, [Ca^2+^], hematocrit and hemoglobin. However, if minimal acceptable values were used instead of optimal values, all measures values bar hematocrit and hemoglobin met the precision targets. We therefore consider the epoc analyzer to be a reasonably precise machine. Unfortunately, no such data is available for the VetStat or cobas b 123 analyzer.

The imprecision of the epoc analyzer for the determination of pCO_2_, pO_2_, and [K^+^] was higher than the pre-set limits of acceptance. The epoc analyzer therefore failed to achieve the required precision for these parameters, while the precision for the remaining parameters was acceptable. This is contradictory to the results obtained in a previous study, where the epoc analyzer met all precision targets except for pCO_2_ [14]. Falsely elevated [K^+^] may arise during sample handling, and although no gross hemolysis was recognized it can not be excluded to have occurred during the consecutive measurements leading to greater variation in the results. The same observation was made for the the cobas b 123 analyzer. Due to technical failure, serial measurements of pO_2_ were not performed with the VetStat analyzer precluding the determination of the precision of this parameter. For the remaining parameters the precision of the VetStat analyzer was considered to be acceptable.

The assessment of precision in this study is limited in part because only 1 blood sample was repeatedly measured, limiting evaluation of the effect of different ranges of values of precision. Furthermore, only the short-time imprecision was assessed. To achieve a more comprehensive assessment of precision, serial measurements of samples with different partial pressures/concentration ranges and over an extended period would be necessary.

The pO_2_ values measured in this study were partly higher than would be expected under usual circumstances due to the partial collection of blood samples under general anesthesia. Therefore, the measured range of pO_2_ values may not represent the expected range for healthy standing unsedated horses. The choice of inclusion of samples under general anesthesia was made in compliance with CLIA guidelines, which suggest inclusion of a wide range of samples [21]. Thus, evaluation of agreement for arterial and venous blood sample measurements would have been beneficial based on different reference ranges expected for arterial versus venous samples. A recent study, evaluating the epoc analyzer using equine arterial and venous blood samples reported the epoc analyzer to have good-to-excellent ability to reliably measure a variety of parameters in both sample types [14]. Another limitation of the present study is that blood protein and lipid concentrations were not assessed. Since protein and lipid may have divergent effects on the accuracy of different measurements certain parameters pending the type of blood gas analyzer used should have been performed in order to assess for any contribution to variability between the 3 analyzers. Sample lipemia or the presence of methylene blue in the sample have been demonstrated to potentially influence the measurement of hemoglobin and the percentage saturation of Hb with O_2_ [30,31]. Unfortunately, hemoglobin and SO_2_ data were not analyzed in the present study. Benzalkonium, a quaternary ammonium substance used as disinfectant, has also been shown to potentially interfere with the measurements of electrolytes, especially [K^+^], [Na^+^] and [Ca^2+^] [32,33]. We excluded this as a potential interference since no benzalkonium-containing disinfectant was used to clean the blood gas analyzers. There was a high variation in [K^+^] measurements in samples analyzed for the within-run precision data of the cobas b 123 and the epoc. Hemolysis is known to increase blood [K^+^] measurements [34] with high degrees of hemolysis also shown to artificially reduce pO2 and increase pCO2 measurements [35]. Although the presence of hemolysis in the samples could not be completely excluded in the present study, the samples were handled with care to minimize hemolysis with no evidence of gross hemolysis in any of the samples.

In conclusion, this study investigated agreement of 2 POC blood gas analyzers against a stationary analyzer which, although widely used in practice, cannot be considered as a gold standard reference. The VetStat analyzer delivered reliable results for the determination of pH, pCO_2_, [HCO_3_^-^] and [K^+^] and may therefore be a useful POC analyzer for equine blood gas analysis. However, measurement of [Na^+^], [Cl^-^] and pO_2_ should be interpreted with caution. The epoc analyzer achieved acceptable results for the determination of [HCO_3_^-^] and [K^+^], while results for pH, pCO2, pO2 and [Na^+^] should be interpreted with caution.
